# Modeling and Measurement of Thermal–Mechanical-Stress-Creep Effect for RF MEMS Switch Up to 200 °C

**DOI:** 10.3390/mi13020166

**Published:** 2022-01-22

**Authors:** Yulong Zhang, Jianwen Sun, Huiliang Liu, Zewen Liu

**Affiliations:** 1 School of Integrated Circuits, Tsinghua University, Beijing 100084, China; zhangyl2020@mail.tsinghua.edu.cn (Y.Z.); sunjw2021@mail.tsinghua.edu.cn (J.S.); 2 Institute of Telecommunication and Navigation Satellites, China Academy of Space Technology, Beijing 100094, China; liuhl15@tsinghua.org.cn

**Keywords:** thermal–mechanical-stress-creep effect, RF MEMS, thermal process, cantilever, package temperature

## Abstract

High-temperature processes, such as packaging and annealing, are challenges for Radio-Frequency Micro-Electro-Mechanical-Systems (RF MEMS) structures, which could lead to device failure. Coefficient of thermal expansion (CTE) mismatch and the material’s creep effect affect the fabrication and performance of the MEMS, especially experiencing the high temperature. In this paper, the Thermal–Mechanical-Stress-Creep (TMSC) effect during thermal processes from room temperature (RT) to 200 °C is modeled and measured, in which an Au-cantilever-based RF MEMS switch is selected as a typical device example. A novel Isolation-Test Method (ITM) is used to measure precise TMSC variation. This method can achieve resolutions of sub-nanometer (0.5 nm) and attofarad (1 aF). There are three stages in the thermal processes, including temperature ramping up, temperature dwelling, and temperature ramping down. In different stages, the thermal–mechanical stress in anchor and cantilever, the grain growth of gold, and the thermal creep compete with each other, which result in the falling down and curling up of the cantilever. These influencing factors are decoupled and discussed in different stages. The focused ion beam (FIB) is used to characterize the change of the gold grain. This study shows the possibility of predicting the deformation of MEMS structures during different high-temperature processes. This model can be extended for material selection and package temperature design of MEMS cantilever in the further studies.

## 1. Introduction

Thermal processes are inevitable in MEMS fabrication. The thermal–mechanical stress and creep are two kinds of typical influences for MEMS structures, which may even result in failure and damage of the MEMS devices. For example, an important thermal process is the packaging, which plays an important role in MEMS devices due to its ability to provide protection and a clean environment for fragile MEMS structures [1,2,3]. The packaging process is always set to be the last step in device fabrication, which means that the MEMS structures must overcome the influences of thermal processes in package, such as mechanical stress, creep, deformation, and so on. The temperature in the packaging processes varies from room temperature (RT) to hundreds of centigrade, normally. As a common factor, temperature determines the influences mentioned above, and the coupling effect of these influences makes things stricter and harsher.

As an example, for a pure gold cantilever-based RF MEMS switch, it is very sensitive to thermal processes because of the characters of the gold material. In the previous studies, thermal–mechanical stress (TMS) effects in RF MEMS switch fabrication processes have been studied systematically, such as the sacrificial layer ashing process [4] and heating process [5]. In [4], the authors investigated the thermal deformations due to the high temperature of ambient plasma, and the temperature distribution caused mechanical stress, making the cantilever curl up significantly. In [5], the authors conducted a thermal–mechanical-stress simulation; and the gold cantilever shows deformation caused by the thermal–mechanical stress. Meanwhile, creep effects caused by viscoelasticity have been also studied in metal MEMS structures, such as microbeam [6], microbridge [7,8], films [9,10,11], laterally actuators [12], and so on. In [6], the authors used a nanoindenter to test the creep of the nickel cantilever. In [7,8], the authors analyzed the creep in a gold RF MEMS switch, and the temperature was used as an accelerating factor to estimate the lifetime of the device. In [9,10], the author characterized the nickel RF-MEMS devices through a highly accurate capacitance-sensing setup under a special bi-state bias condition, and the model reveals that the creep deformation is dominated by Coble creep. In [11], the authors focus on the creep performance of gold and evaluated the uniaxial Au tensile specimens by a special MEMS structure, and their work demonstrates a powerful drift-free nanomechanical test platform for mechanical property measurement of freestanding thin films subjected to uniaxial tension. In [12], the authors conducted the analysis of thermomechanical coupling damage behavior in the long-term performance of MEMS actuators. The proposed damage model and numerical method can provide effective assessment in the long-term performance of MEMS thermal actuators. However, there are few studies combining thermal–mechanical stress and thermal-creep (TC) effect, which is a limitation for MEMS devices design and fabrication. In this paper, we introduce the Thermal–Mechanical-Stress-Creep (TMSC) coupling model into MEMS devices, and the gold-cantilever-based RF MEMS switches are selected as an example for the study.

Meanwhile, in the previous studies, the methods for the test of the thermal–mechanical-stress effects and creep effects in MEMS structures include direct methods, such as microtension [11,13,14], nanoindentation [6,15], and indirect methods, such as optical curvature [4,16,17], voltage [18,19,20], capacitance [9,10,21], and so on. However, these methods are not suitable for DC-contact RF MEMS switches. In this study, we use a novel method, which is called the in situ Isolation-Test Method (ITM), to measure the tiny variations in the switches.

This paper is organized as follows. In Section 2, the TMSC effect model of RF MEMS switch is introduced and developed, including the thermal–mechanical-stress effect and the thermo-creep effect. In Section 3, the gold-cantilever-based RF MEMS switch is designed and fabricated. In Section 4, the ITM is developed for the in situ measurement of cantilever deformation. In Section 5, the TMSC effect is tested carefully. The test result is analyzed and discussed in this section, and the TMSC model is verified and decoupled. Finally, in Section 6, we conclude this investigation, and further study is proposed to improve the TMSC model and the performance of the RF MEMS switch.

## 2. TMSC Model

**Figure 1:**
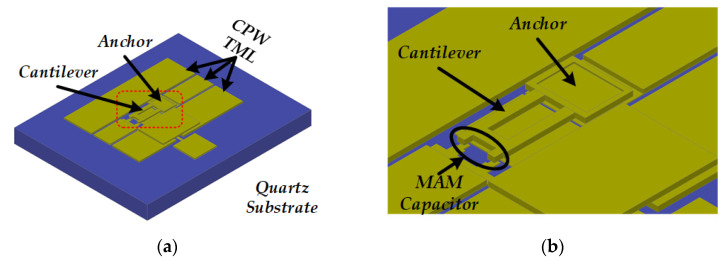
Schematic diagram of the gold-cantilever-based RF MEMS switch: (**a**) Overview of the device, (**b**) Close-up view of the anchor, cantilever, and MAM capacitor.

The gold-cantilever-based RF MEMS switch is illustrated in Figure 1. The device consists of several parts, which are listed below:

1. The anchor that is fabricated on the substrate, with width *W* and thickness *t*;
2. The cantilever that may fall down or curl up, with length *L_can_*;
3. The metal–air–metal (MAM) capacitor, with gap;
4. RF coplanar waveguide (CPW) transmission line (TML).

The TMSC model of the device includes two parts: Thermal–Mechanical-Stress (TMS) effect and Thermal-Creep (TC) effect. They work in different parts and different temperatures, as follows.

### 2.1. Thermal–Mechanical-Stress Effect

#### 2.1.1. Thermal–Mechanical-Stress Effect in Anchor

**Figure 2:**
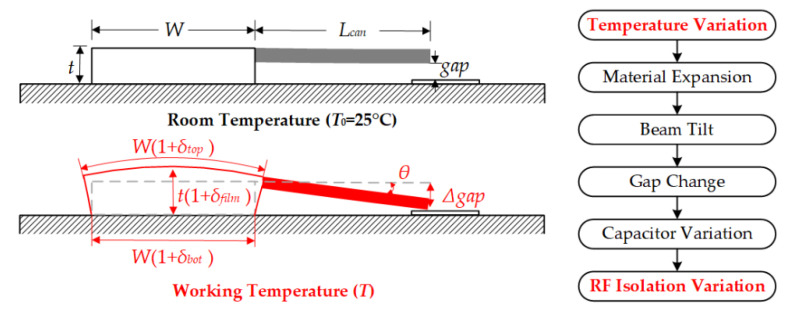
Schematic diagram of the Thermal–Mechanical-Stress Effect caused by thermal mismatch in RF MEMS anchor structure, with situations of Room Temperature (*T*_0_) and Working Temperature (*T*).

The cross-section schematic diagram of the RF MEMS structure is shown in Figure 2, in which the situations of Room Temperature (*T*_0_) and Working Temperature (*T*) are both displayed. When the temperature increases, the materials in the structure will extend, including the substrate, film, and cantilever. The top surface width and the bottom surface width of the film will change to be *W*(1 + *δ_top_*) and *W*(1 + *δ_bot_*), respectively. Meanwhile, the thickness of the film will become *t*(1 + *δ_film_*). As a result of the deformation of the film, the beam will tilt with *θ* along the tangential direction. As a result, the gap and the capacitance (*C_u_*) of the MAM capacitor change. Finally, the isolation of the RF MEMS structure will vary with the change of temperature.

According to the geometrical relationship, it is easy to obtain three equations, just as shown in (1), in which *θ*(*T*) and *A*(*T*) are temperature-dependent coefficients.

(1) $${\Delta gap}_{anchor}=\theta \left(T\right)\times L_{can}\times \left(1+\delta_{film}\right)=A\left(T\right)\times L_{can}$$

**Figure 3:**
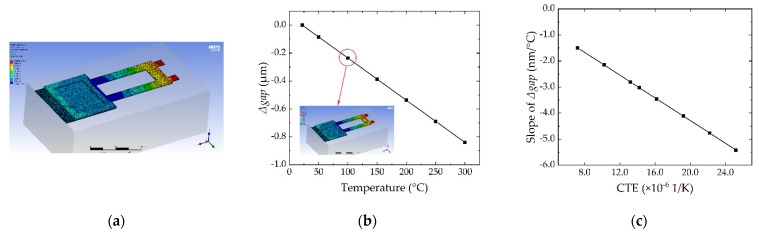
Simulation results for TMS effect of the RF MEMS device: (**a**) Deformation of the device heated up to 100 °C; (**b**) Simulated *Δgap* versus temperature when the CTE of gold is 14.2 × 10^−6^ 1/K; (**c**) Simulated slope of *Δgap* versus CTE.

The TMS effect in anchor simulation is conducted by Finite Element Analysis (FEA) software ANSYS. Figure 3a shows the simulated result when the device is heated up to 100 °C. The maximum deformation (red color) appears in the tip of the cantilever, which is the focus of *Δgap*. In Figure 3b,c, the *Δgap* varies linearly with temperature, and the slope of *Δgap* varies linearly with CTE, which is caused by the linear expansion simulation setting. In fact, the relationship of *Δgap* and temperature is not a perfect linear relationship, and they will show some variation performance, which will be shown in the following evaluation.

#### 2.1.2. Thermal–Mechanical-Stress Effect in Cantilever

**Figure 4:**
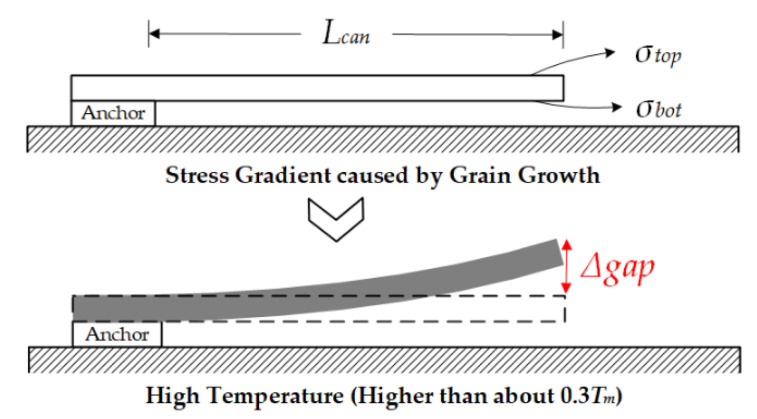
Schematic diagram of the Thermal–Mechanical-Stress Effect caused by the grain growth of gold in the RF MEMS structure.

The thermal–mechanical-stress effect not only works in the anchor but also in the cantilever. As a result of the fabricating process, there are many ultrafine crystalline grains in the cantilever [22]. When the device is heated, especially when the temperature reaches about 30% of the melting point (0.3*T_m_*) [23], it will cause growth of the grains, as shown in Figure 4. The stresses on the top and bottom surfaces are *σ_top_* and *σ_bot_*, respectively. Owing to the stress gradient, the cantilever will show curling-up performance, which can be described by (2) and (3), in which *Γ*(*T*) is the strain gradient related to temperature, and *E* is the Young’s Modulus of the cantilever material.

(2) $$\sigma_{\Delta }=\sigma_{top}-\sigma_{bot}=E\Gamma \left(T\right)z$$

(3) $${\Delta gap}_{can}=\frac{\Gamma \left(T\right)L_{can}^{2}}{2}$$

**Figure 5:**
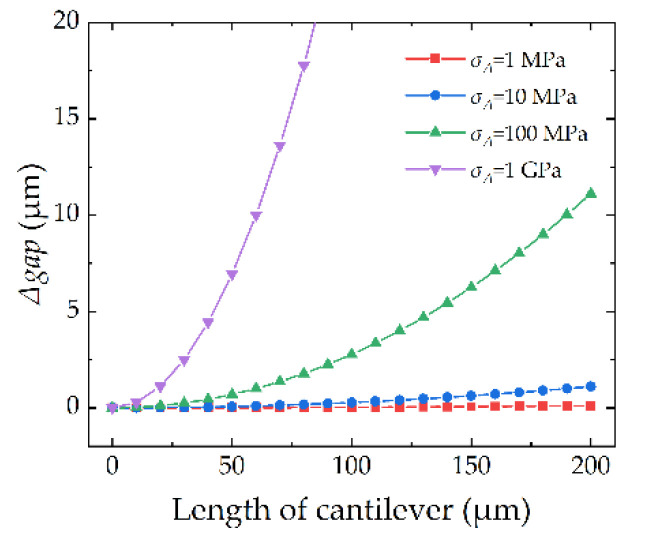
Calculated *Δgap* versus length of cantilever for different stress *σ_Δ_*.

The calculated gap change caused by the TMS effect in the cantilever is shown in Figure 5. When the stress in cantilever *σ_Δ_* is higher, the *Δgap* will be more significant. So, we can predict that for a thermal process with higher temperature, the cantilever will show more curling-up performance due to the bigger gold grain. Meanwhile, the grain growth not only depends on temperature but also time. When it is heated to a higher temperature, it will need a shorter time to finish the grain growth. This will be verified in the following measurement.

### 2.2. Thermal-Creep Effect

**Figure 6:**
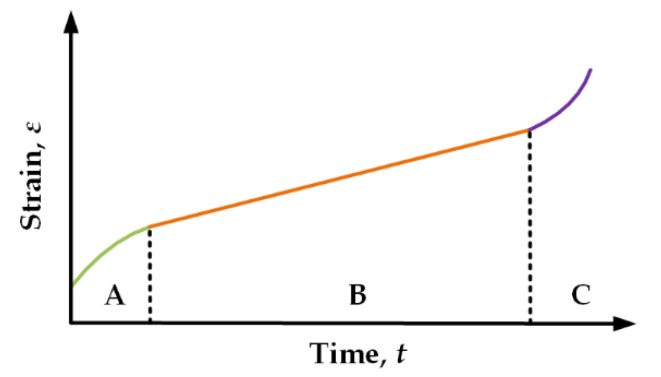
Creep curve of the materials with viscoelasticity performance.

Figure 6 shows the typical creep curve of the materials with viscoelasticity performance under the situation of constant temperature and constant stress [24]. Normally, there are three stages of the creep curve: transient creep (Stage A), steady-state creep (Stage B), and accelerating creep (Stage C). During Stage A, the slope of strain is a monotonically decreasing function; during Stage B, the slope of strain is a constant, and the time of Stage B is much longer than that of Stages A and C, which makes Stage B the most important stage in creep phenomena; during Stage C, the strain rate increases sharply and may lead to rupture of the materials. For the RF MEMS switch, the gap of the MAM capacitor is always several micrometers, and the deformation of the cantilever is also about several micrometers. So, Stage C is out of our sight. With the consideration of negligible short time of Stage A, we focus on Stage B in this paper.

When temperature increases, thermal–mechanical stress will appear in the device. Due to the length of the constant temperature period, the stress will cause creep of the cantilever because of the gold viscoelasticity performance. For ultrafine-grain-rich electroplated gold, grain boundary diffusion is considered as the main factor of the creep. As one of the most important creep types of diffusion creep, Coble creep [25] can be described by (4).

(4) $$\varepsilon^{\prime }=A\frac{D_{g0}\delta \Omega \sigma }{\pi d^{3}kT}\exp\left(-\frac{Q_{g}}{kT}\right)$$

in which *εʹ* is the strain rate, σ is the applied stress, *D_g_*_0_ is the diffusivity constant, *Q_g_* is the activation energy, *δ* is the thickness of grain boundaries, *Ω* is the atomic volume, *d* is the grain size, *k* is Boltzmann’s constant, and *T* is temperature.

The parameters above for materials are different with each other. They are strongly influenced by the fabrication processes. So, the simulation for TC effect is not conducted, and the *Δgap_c_* (creep-caused *Δgap*) of the device is mainly illustrated by the test results shown in Section 5.

### 2.3. TMSC Model

With all the considerations of Thermal–Mechanical-Stress effect and Thermal-Creep effect, we can calculate the change of *gap* (*Δgap*) as (5).

(5) $$\Delta gap\left(T,t\right){=\Delta gap}_{anchor}{+\Delta gap}_{can}{+\Delta gap}_{c}{+\Delta gap}_{else}$$

in which *Δgap_anchor_* is the variation caused by thermal mismatch in the anchor, *Δgap_can_* is the variation caused by stress in the cantilever, *Δgap_c_* is the variation caused by creep, and *Δgap_else_* is the variation caused by other reasons, such as electrostatic force and so on. During the thermal processes, the *Δgap_else_* can be ignored owing to the absence of other factors.

Moreover, in different thermal stages, things are different. For example, during the stage of temperature ramping up and ramping down, *Δgap_anchor_* is the main factor; when the temperature is higher than 0.3*T_m_*, *Δgap_can_* is the main factor; and when the device is staying at a constant temperature, *Δgap_c_* becomes the main factor. These will be shown, verified, and decoupled in the following sections.

## 3. RF MEMS Device Design and Fabrication

**Figure 7:**
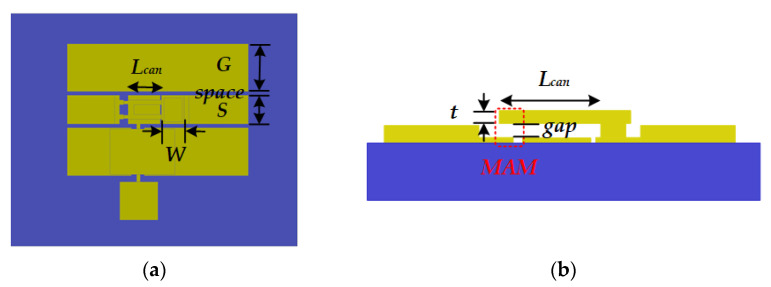
(**a**) Top view and (**b**) Cross-section view of the RF MEMS device with an MAM capacitor.

The top view and cross-section view of the RF MEMS device with a MAM capacitor are illustrated in Figure 7. The designed parameters of the RF MEMS switch are shown in Figure 5 and Table 1, in which the lengths of the cantilever (*L_can_*) are designed to be 80–160 μm, the thickness of the cantilever (*t*) is set to be ≈2.5 μm, and the gap of the MAM capacitor is set to be about 1 μm.

**Figure 8:**
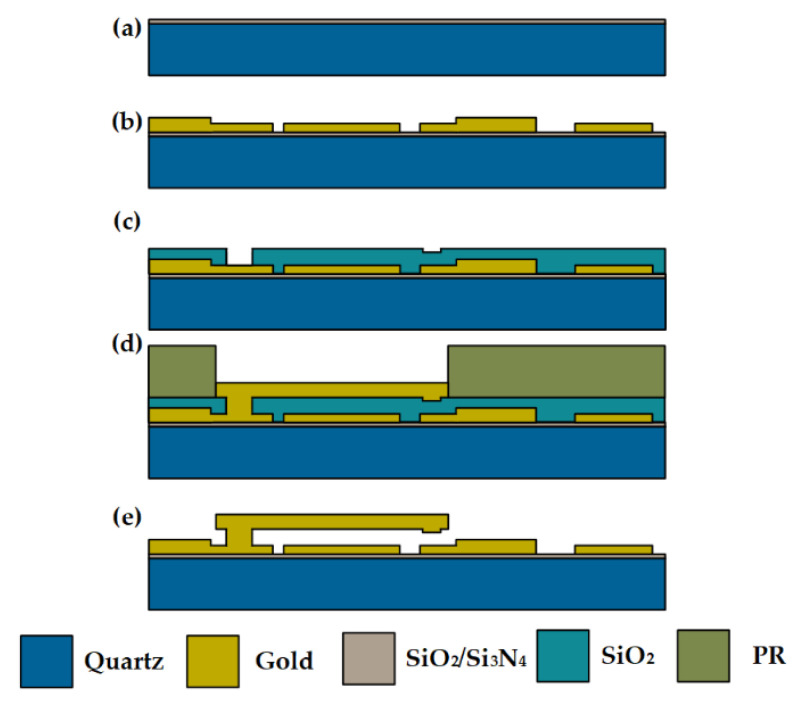
Process flow of the RF MEMS device.

The devices are fabricated by using MEMS surface manufacturing processes, as shown in Figure 8: (a) Quartz is selected as substrate; SiO_2_ and Si_3_N_4_ are deposited as a buffer layer and etch-protection layer; (b) CPW TML is fabricated by sputtering and electroplating to obtain two different thickness gold layers; (c) A SiO_2_ sacrificial layer is deposited by using PECVD, and the contact dimple and anchor hole are etched; (d) A gold cantilever is electroplated; (e) A sacrificial layer is removed by wet etching, and a critical point dryer is employed to prevent the adhesion of the cantilever. Then, the fabrication process is finished.

**Figure 9:**
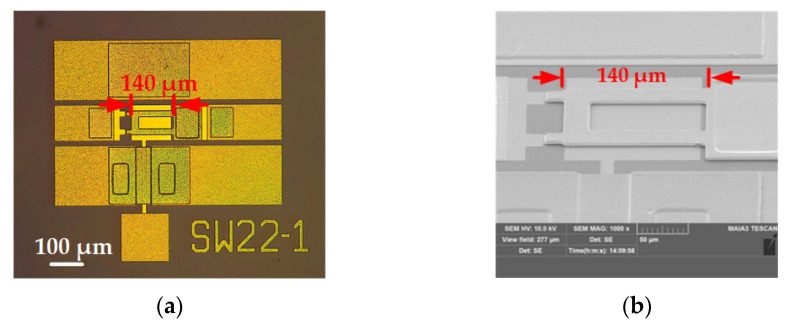
(**a**) OM and (**b**) SEM photos of the RF MEMS device.

Before the test, the switches are photographed by optical microscope (OM) and scanning electron microscope (SEM), as shown in Figure 9, in which the device with 140 μm length and 2.79 μm thicknesses cantilever is selected as a representative of these switches.

## 4. Isolation-Test Method

**Figure 10:**
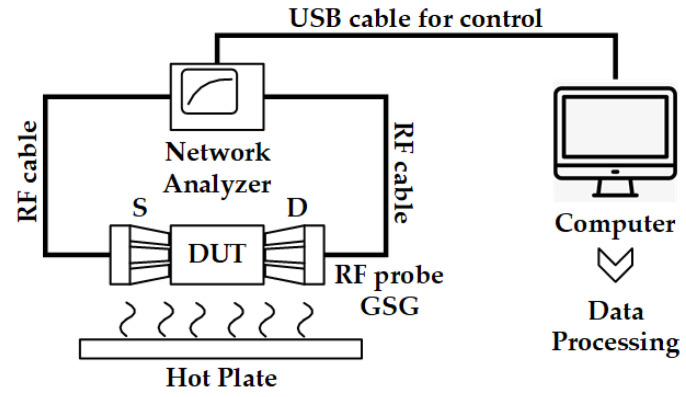
The test system of in situ Isolation-Test Method for a long thermal process test.

The test system is illustrated in Figure 10. The device under test (DUT) is put on the hot plate of the RF probe station Cascade Summit 12,000 M. The network analyzer Keysight N5290A is used to measure the S-Parameters of the device, especially isolation included. A computer is employed to control the network analyzer. With the help of a computer, the S-Parameters are recorded automatically with a fixed interval, such as per ten seconds. Then, the S-Parameters are processed by a data processing program.

**Figure 11:**
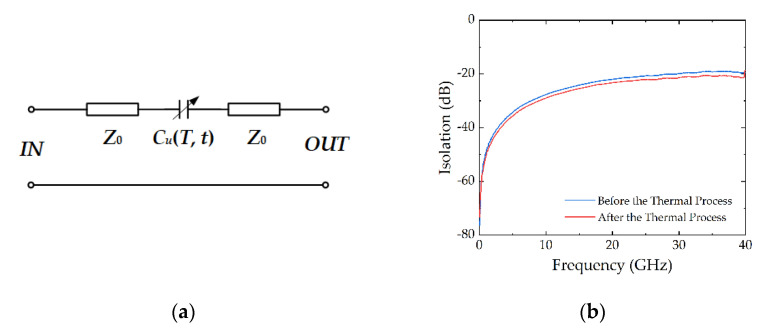
(**a**) Equivalent circuit model of the RF MEMS structure, in which the capacitor is variable at different working temperatures and different times; (**b**) Isolation performance of the device before and after the thermal process.

The equivalent circuit model of the RF MEMS device is shown in Figure 11a, in which the capacitor is variable at different working temperatures and different times. Its isolation performances are plotted in Figure 11b, including the status before and after the thermal process. It is noted that the change in the isolation of the switch before and after the thermal process is small. However, the changes of capacitance *C_u_* and *gap* cannot be negligible, owing to the relatively big change in *gap*.

The isolation of the device can be calculated by (6). On the basis of (6), we can calculate the MAM capacitance (*C_u_*) by the least square method (LSM). The detailed information of the LSM is shown in Appendix A, and the capacitance *C_u_* is calculated by the frequency from 50 MHz to 20 GHz but not by a single frequency point.

(6) $${Iso=S}_{21}\approx \frac{j2{\omega C}_{u}Z_{0}}{1+j2{\omega C}_{u}Z_{0}}$$

The MAM capacitance (*C_u_*) is related to the gap of the MAM capacitor, as illustrated in (7), in which *A* is a coefficient related to the plate capacitance and fringe capacitance, and *A* is related to the *gap* [26]. It is noted that the *gap* of MAM capacitor is not a constant, so we use the effective gap (*gap_eff_*) and generalized exponential function to describe the relationship of *gap_eff_* and *C_u_*.

(7) $$C_{u}=A\frac{{\varepsilon S}_{MAM}}{{gap}_{eff}}=\alpha \cdot {gap}_{eff}^{\beta }$$

**Figure 12:**
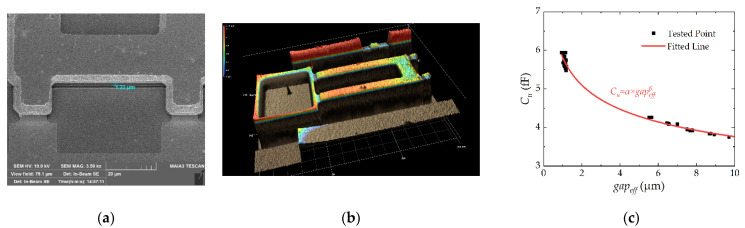
(**a**) The *gap* tested by SEM; (**b**) The gap tested by 3D optical microscope; and (**c**) The relationship of *gap_eff_* and *C_u_* calibrated by ITM, SEM, and optical microscope.

For precise test, the direct test method SEM and 3D optical microscope are used to calibrate the ITM. As shown in Figure 12, α is 5.8112 fF and β is −0.18875. Then, the tested *gap_eff_* can be obtained by interpolation between *C_u_* and *gap_eff_*.

**Figure 13:**
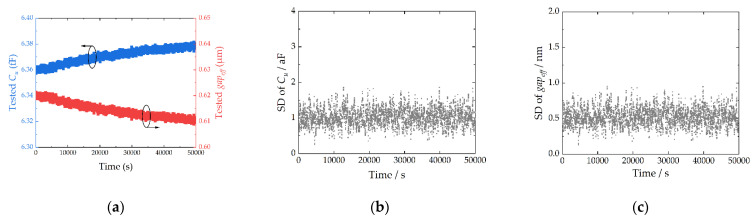
Performance of the ITM during 50,000 s: (**a**) The tested *C_u_* and *gap_eff_*; (**b**) Standard deviation of *C_u_*; (**c**) Standard deviation of *gap_eff_*.

The resolution of ITM is measured, and the test system shows a stable uncertainty, as shown in Figure 13. The standard deviation (SD) of *C_u_* is about 1 aF, and the SD of *gap_eff_* is about 0.5 nm, which are calculated by 10 samples. The resolutions are excellent for the TMSC test.

## 5. TMSC Effect Measurement and Discussion

### 5.1. Temperature in Thermal Processes

**Figure 14:**
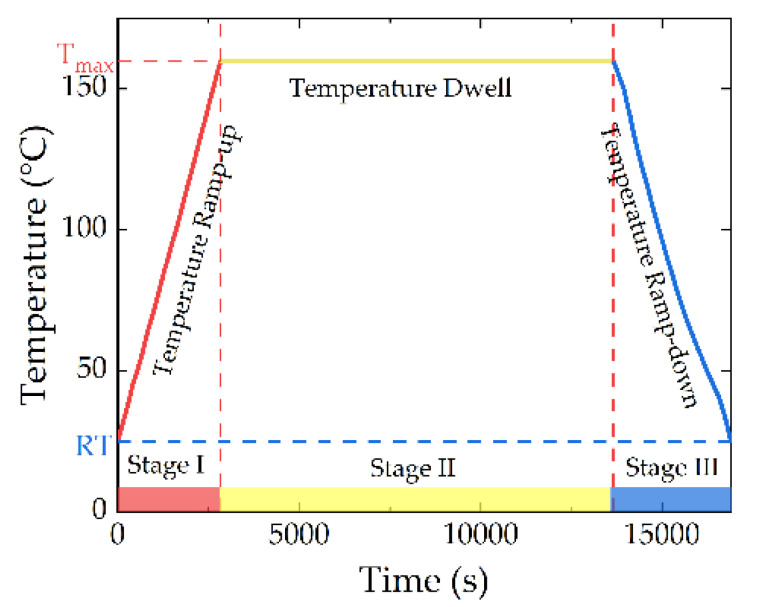
Three stages of the thermal process, including Temperature Ramping Up (Stage I), Temperature Dwelling (Stage II), and Temperature Ramping Down (Stage III). (160 °C process is selected as an example).

The thermal process consists of three stages, and they are temperature ramping-up stage (Stage I, red color), temperature-dwelling stage (Stage II, yellow color), and temperature ramping-down stage (Stage III, blue color), as shown in Figure 14. The RT 160 °C process is selected as an example. It takes about one hour to reach the maximum temperature (*T_max_*) during Stage I, and it lasts three hours during Stage II; finally, it takes about another one hour to ramp down to RT during Stage III.

### 5.2. Response of the Device during Different Thermal Processes

**Figure 15:**
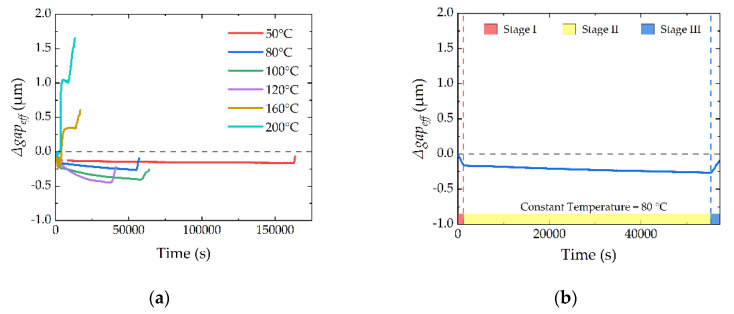

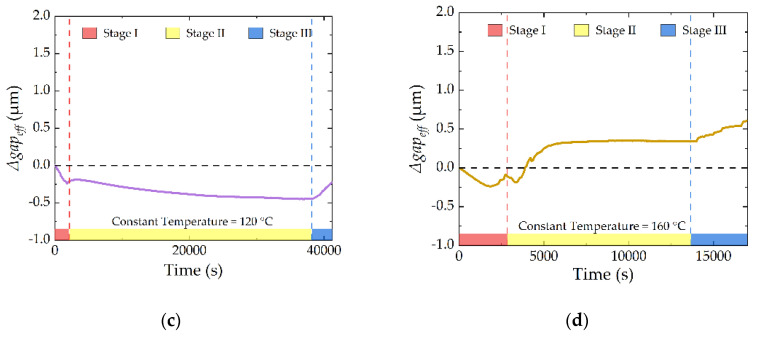
Thermal responses of the device. (**a**) *Δgap_eff_* during different thermal processes, including 50–200 °C; (**b**) *Δgap_eff_* of the thermal process from RT to 80 °C to RT; (**c**) *Δgap_eff_* of the thermal process from RT to 120 °C to RT; (**d**) *Δgap_eff_* of the thermal process from RT to 160 °C to RT.

The thermal responses of the device with 140 μm-length cantilevers are plotted in Figure 15. Figure 15a includes all the different thermal processes from 50 to 200 °C. For more detailed information, the thermal processes of 80, 120, and 160 °C are plotted in Figure 15b–d, respectively. There is a conclusion that the devices show different performances among different temperature processes. For 50 to 120 °C, the devices show falling-down performance, and when the temperature is higher, the falling down of the cantilever is more evident. However, for 160 to 200 °C, things are different. The device shows curling-up performance, and when the temperature is higher, the curling up of the cantilever is more evident. The performance verifies the model established in Section 2, and more details will be discussed as follows.

#### 5.2.1. Stage I: Temperature Ramping Up

**Figure 16:**
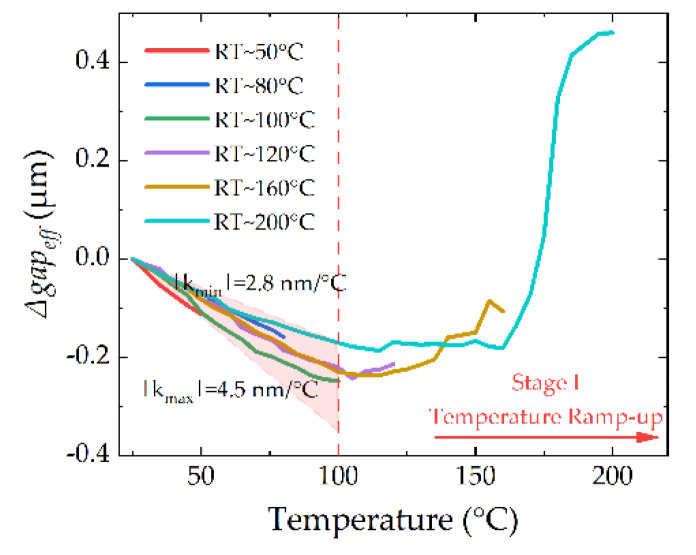
Thermal responses of the device during Temperature Ramping-Up stage (Stage I).

The changes of the *gap_eff_* in Stage I are plotted in Figure 16. Obviously, there is a turning point after 100 °C. Before 100 °C, the cantilevers fall down due to the Thermal–Mechanical-Stress Effect in the anchor, and the slopes of *Δgap_eff_* are between 2.8 and 4.5 nm/°C. After 100 °C, the grain growth of gold competes with the thermal mismatch in the anchor, and the grain growth defeats the thermal mismatch finally. The cantilever shows curling-up performance, and when the temperature is higher, the device shows more curling-up performance.

For the turning point, it is strongly correlated with the fabrication process and grain situation. As a rule of thumb, the annealing and recrystallization temperature for gold is in the range of 150–200 °C [23]. Even some reports show that pure gold may show recovery, grain growth, and recrystallization at ambient temperatures [23,27,28]. In this paper, the grain growth temperature is about 100 °C, which is highlighted in Figure 16.

The grain growth also depends on time. If the temperature is higher, the time needed to finish growth is shorter. The growth in the 200 °C process finishes in Stage I, and the *Δgap_eff_* reaches a stable value after grain growth, as shown in Figure 16. However, things are different for the 160 °C process. Compared with the 200 °C process, the grain growth in the 160 °C process even lasts about 30 min in Stage II, as shown in Figure 15d.

To reduce the changes of the *gap* in Stage I, there are several methods inspired by Figure 16 and Section 2.1. The coefficient of thermal expansion (CTE) of the substrate and cantilever need to match with each other as best as possible. The size of the anchor needs to be optimized to reduce the effect of thermal mismatch, and the grain growth temperature of the cantilever material needs to be as high as possible.

It is noted that the curve of 200 °C process in Figure 16 is retested by another switch because of the unstable performance in Figure 15a, and this is the reason why the highest *Δgap_eff_*s in Figure 15a and Figure 16 are different.

#### 5.2.2. Stage II: Temperature Dwelling

**Figure 17:**
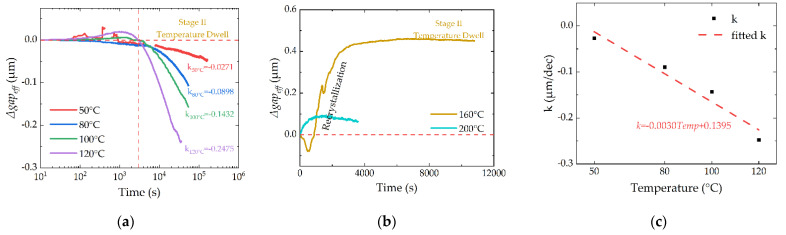
(**a**) Thermal responses of the device during Temperature Dwelling stage (Stage II) for 50 to 120 °C; (**b**) Thermal responses of the device during Temperature Dwelling stage (Stage II) for 160 and 200 °C; (**c**) Change speeds of the cantilevers for 50 to 120 °C.

The thermal responses of the device during Stage II are illustrated in Figure 17.

The changes of the *gap_eff_* for 50 to 120 °C in Stage II are plotted in Figure 17a. Since the main influencing factor for 160 to 200 °C is different with low temperature, they are plotted separately in Figure 17b. For Figure 17a,b, the *Δgap_eff_* is modified. The beginning state of Stage II is selected to be the zero line.

The responses in Figure 17a,b are different. The main influencing factor of Figure 17a is the thermal creep of the gold material. The *Δgap_eff_* follows an exponential function with time, so the x-axis in Figure 17a is plotted in a log-scale. The change speeds of the cantilevers are strongly influenced by temperature, as shown in Figure 17c, which is consistent with the Cobel creep theory. This can be used to predict the lifetime of the RF MEMS device, just as in Refs. [7,8,29]. The lifetime of this gold RF MEMS switch under 80 °C is about 13 days, which is too short to satisfy the need for electronic systems. The special materials with better thermal-creep performance need to be assessed for a higher lifetime.

The main influencing factor of Figure 17b is grain growth. At 160 and 200 °C, the grains of the cantilever reach a stable state, and the thermal–mechanical stress in the device is released by the grain growth. So, there is no significant creep phenomenon. The x-axis in Figure 17b is plotted in a linear-scale, which is different with Figure 17a.

#### 5.2.3. Stage III: Temperature Ramping Down

**Figure 18:**
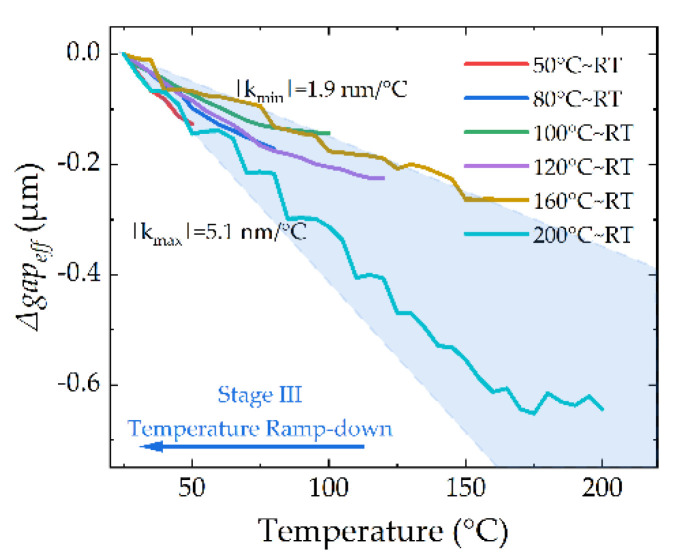
Thermal responses of the device during the Temperature Ramping-Down stage (Stage III).

The changes of the *gap_eff_* in Stage III are plotted in Figure 18, and the *Δgap_eff_* is also modified. The end state of Stage III is selected as the zero line. During the Temperature Ramping-Down stage, the cantilevers recover back due to thermal mismatch recovery in the anchor, and the slopes of *Δgap_eff_* are between 1.9 and 5.1 nm/°C.

The slope ranges of *Δgap_eff_* in Stage III are slightly different from Stage I; they are 1.9–5.1 nm/°C and 2.8–4.5 nm/°C, respectively. We think that this is influenced by the change of the gold grains due to grain growth.

### 5.3. Comparison of the Device before and after the Thermal Processes

**Figure 19:**
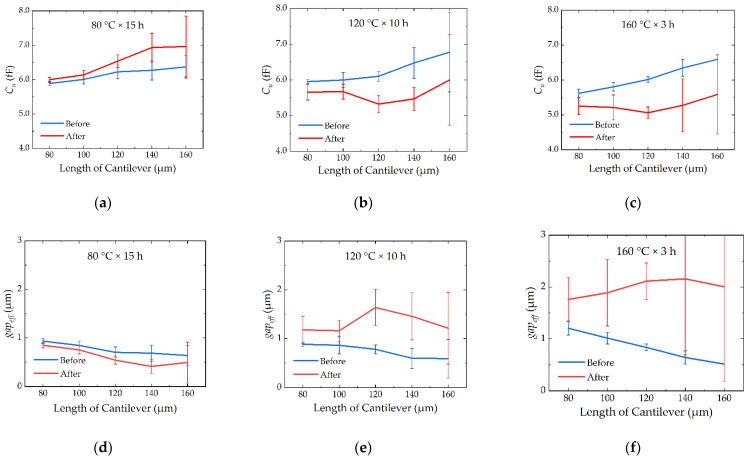
Comparison of the (**a**–**c**) *C_u_* and (**d**–**f**) *gap_eff_* before and after the thermal processes.

Comparisons of the MAM capacitance *C_u_* and *gap_eff_* before and after the thermal processes are shown in Figure 19, with 80 °C, 120 °C, and 160 °C included. Different cantilever lengths are plotted. Before the thermal processes, the *C_u_* and *gap_eff_* of the devices show no difference from each other. However, after the different thermal processes, they show different curling-up or falling-down performances. We can draw some conclusions: for the 80 °C process, the device is mainly influenced by thermal creep of gold; for the 160 °C process, the device is mainly influenced by the grain growth of gold; for the 120 °C process, the two factors compete with each other, and the thermal–mechanical stress in the anchor caused by thermal mismatch works in every thermal process.

**Figure 20:**
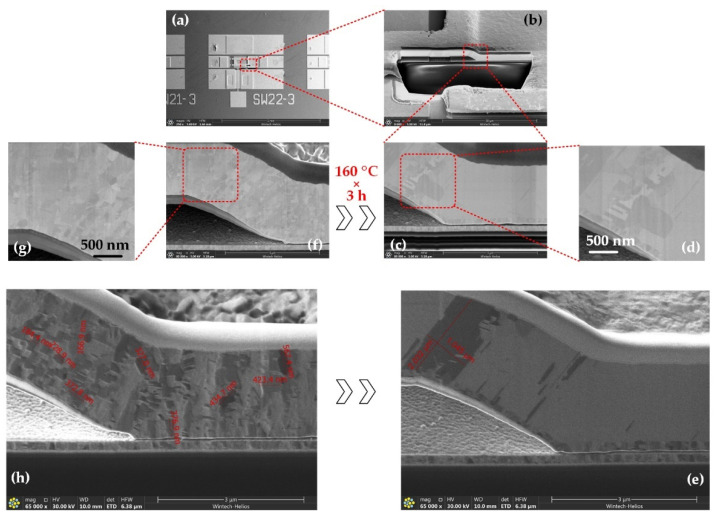
Photos of the device by FIB and SEM: (**a**–**e**) After three hours 160 °C thermal process; (**f**–**h**) Without any thermal process.

For more details of the device before and after thermal processes, focused ion beam (FIB) and SEM are used to disclose the change in the cantilever, as shown in Figure 20. Figure 20f–h is the device without any thermal processes, and (a–e) is the device after about three hours in the 160 °C annealing process. It is clear that the gold grains change significantly, which shows the effect of gold grain growth. The average grain size grows from about 300 nm to bigger than 2.0 μm, as shown in Figure 20e,h.

### 5.4. Summary

On basis of the test results above, the main influencing factors of the thermal processes are listed and decoupled in Table 2. It is noted that the first column of Table 2 is the maximum temperature (*T_max_*) of the thermal processes. It represents a thermal process (RT~*T_max_*~RT) but not a single temperature point.

During Stage I of Temperature Ramping Up, from RT to 100 °C, the thermal–mechanical stress in the anchor is the main influencing factor, which will cause falling down of the cantilever; from 160 to 200 °C, the thermal–mechanical stress caused by the grain growth of gold will result in curling up of the cantilever; from 100 to 160 °C, the two factors compete with each other, which will result in an unstable state of falling down or curling up.

During Stage II of Temperature Dwelling, from RT to 100 °C, the thermo creep (Cobel creep) will result in falling down of the cantilever; from 160 to 200 °C, the cantilever will stay at a steady state owing to the grown grain.

During Stage III of Temperature Ramping Down, the thermal–mechanical stress in the anchor will cause the recovery of the cantilever.

### 5.5. Generalization of the Method

**Figure 21:**
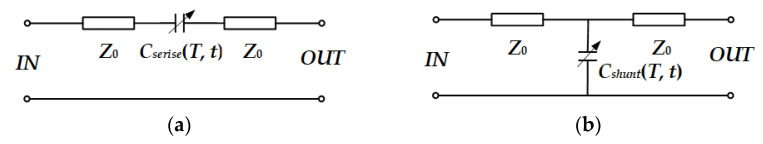
Equivalent circuit models of (**a**) Series capacitor and (**b**) Shunt capacitor in RF MEMS switches.

According to the working mode, there are two kinds of capacitor in RF MEMS switches, which can be called the series capacitor and shunt capacitor, respectively, as shown in Figure 21.

The isolation (S_21_) of devices based on the series capacitor is illustrated by (8), and the return loss (S_11_) of devices based on the shunt capacitor is illustrated by (9). On the basis of (8) and (9), the capacitance in RF MEMS switches can be calculated.

(8) $${Isolation=S}_{21}\approx \frac{j2{\omega C}_{\mathrm{serise}}Z_{0}}{1+j2{\omega C}_{serise}Z_{0}}$$

(9) $${Return\;Loss=S}_{11}\approx \frac{-{j\omega C}_{shunt}Z_{0}}{{2+j\omega C}_{shunt}Z_{0}}$$

Moreover, the developed method is not only suitable for a single capacitor but also suitable for a complex capacitor network, which means that this method can be generalized to other types of RF MEMS switches such as clamped–clamped bridges or laterally actuated RF MEMS switches.

## 6. Conclusions

The TMSC effect is introduced in this paper. A gold-cantilever-based RF MEMS switch is selected as a typical device to model and verify the TMSC effect. The ITM is used to measure the tiny deformation caused by TMSC. The test results show that thermal–mechanical stress and thermal creep (Cobel creep) compete with each other at different temperatures, which results in curling up and falling down of the cantilever. The temperature of 100 °C is an important turning point for the influencing factor changing after 100 °C. The change of gold grain is one of the key reasons for the deformation. This gives us the inspiration of overcoming or reducing the TMSC effect.

Furthermore, the TMSC model can be used to design the thermal process and estimate the influence of different thermal processes. To weaken the TMSC effect and improve the reliability of the RF MEMS switch, there are two promising ways: low-stress cantilever design and strengthened material design. Chamfering and thick cantilever design can be considered for low stress design. Hard metals (Ni, Cu) and alloys (Au-Ni alloy, Au-Cu alloy, Ni-W alloy) are the candidates for the strengthened material design. This developed TMSC model can be extended for evaluation of the two ways in further studies, which is a great need for RF MEMS devices.

## Figures and Tables

**Table 1:** Designed parameters of the device

Part	Symbol	Description	Value (μm)
CPW TML	*L_TML_*	length of the CPW TML	750
	*G*	width of the ground line	200
	*space*	space between G and S	16
	*S*	width of the signal line	120
Anchor	*W*	width of the anchor	75
	*t*	thickness of the anchor	≈2.5
Cantilever	*L_can_*	length of the cantilever	80~160
	*t*	thickness of the cantilever	≈2.5
	*w_beam_*	width of the beam in cantilever	25
MAM Capacitor (Contact)	*l_contact_*	size of the contact	8
	*gap*	gap of the MAM	≈1

**Table 2:** Influencing factors of the TMSC model during different stages.

Max Temperature (*T_max_*) of the Thermal Processes	Stage I Temperature Ramping Up	Stage II Temperature Dwelling	Stage III Temperature Ramping Down
RT–100 °C	Thermal–Mechanical Stress	Thermal Creep	Thermal–Mechanical Stress
100–160 °C	Thermal–Mechanical Stress Grain Growth	Thermal Creep Grain Growth	Thermal–Mechanical Stress
160–200 °C	Thermal–Mechanical Stress Grain Growth	Grain Growth	Thermal–Mechanical Stress

## Appendix A

The MAM capacitance (*C_u_*) is calculated by the least square method (LSM), and the data from 50 MHz to 20 GHz are used, but not by a single frequency point, just as follows.

First, we rewrite (6) as (A1).

(A1) $${Iso=S}_{21}\approx \frac{j2{\omega C}_{u}Z_{0}}{1+j2{\omega C}_{u}Z_{0}}$$

The |*S*_21_|^2^ can be expressed as (A2), according to (A.1).

(A2) $${\left|S_{21}\right|}^{2}\approx {\left|\frac{j2\omega C_{u}Z_{0}}{1+j2\omega C_{u}Z_{0}}\right|}^{2}=\frac{4\omega^{2}C_{u}^{2}Z_{0}^{2}}{1+4\omega^{2}C_{u}^{2}Z_{0}^{2}}$$

Then, we can derive the expression as (A3).

(A3) $$4\omega^{2}Z_{0}^{2}\left(1-{\left|S_{21}\right|}^{2}\right)C_{u}^{2}={\left|S_{21}\right|}^{2}$$

We introduce *α* and *β* as (A4) and (A5) to simplify (A3).

(A4) $$\alpha =4\omega^{2}Z_{0}^{2}\left(1-{\left|S_{21}\right|}^{2}\right)$$

(A5) $$\beta ={\left|S_{21}\right|}^{2}$$

Then, (A3) can be simplified as (A6).

(A6) $$\alpha C_{u}^{2}=\beta$$

We define the inner product of *α* and *β* as (A7), in which *α* and *β* are arrays with N elements from 50 MHz to 20 GHz.

(A7) $$\left(\alpha ,\beta \right)=\sum_{i=1}^{N}\alpha_{i}\beta_{i}$$

Finally, we use the LSM to solve (A3) to calculate the C_u_ as (A8) and (A9).

(A8) $$C_{u}^{2}=\frac{\left(\alpha ,\beta \right)}{\left(\alpha ,\alpha \right)}$$

(A9) $$C_{u}=\sqrt{\frac{\left(\alpha ,\beta \right)}{\left(\alpha ,\alpha \right)}}$$

Based on the calculation above, we can obtain the changed *C_u_* from the small changed isolation. Then, the gap can be calculated by the relationship of *C_u_* and *gap*.
